# Primary Acquired Toxoplasma Retinochoroiditis: Choroidal Neovascular Membrane as an Early Complication

**DOI:** 10.7759/cureus.4001

**Published:** 2019-02-04

**Authors:** Faiza Mushtaq, Amna Ahmad, Fizza Qambar, Aysha Ahmad, Naveen Zehra

**Affiliations:** 1 Ophthalmology, Dow University of Health Sciences, Karachi, PAK; 2 Internal Medicine, Dow University of Health Sciences, Karachi, PAK

**Keywords:** ocular toxoplasmosis, acquired toxoplasmosis, toxoplasma retinochoroiditis, choroidal neovascular membrane (cnvm), anti-vascular endothelial growth factor (anti-vegf)

## Abstract

Ocular toxoplasmosis occurs subsequently after systemic infection with the protozoan parasite, Toxoplasma gondii (T. gondii). The parasite has a high affinity for retinal microvascular endothelium with the retina being the primary site of infection in the eye. Choroidal neovascular membrane (CNVM) is a late complication of ocular toxoplasmosis, mostly occurring in healed, inactive lesions and may be a cause of sudden loss of vision, especially in young patients. However, we report a case of a 22-year-old female who presented to our clinic with CNVM as an early complication. She complained of metamorphopsia and diminished vision in her right eye. Ocular examination, serological investigation and fundoscopy, fundus fluorescein angiography (FFA), axial optical coherence tomography (OCT), and optical coherence tomography angiography (OCTA) were carried out and a diagnosis of primary acquired Toxoplasma retinochoroiditis with active CNVM was made. Treatment was commenced with sulfamethoxazole and trimethoprim. Oral prednisolone and intravitreal injection of the anti-vascular endothelial growth factor (anti-VEGF), bevacizumab, were also given. This report describes the rare presentation of ocular toxoplasmosis as a primary lesion in which adjacent pre-existing fundal scarring was absent. The lesion had an acquired etiology in an immunocompetent patient and was complicated early by CNVM.

## Introduction

Ocular toxoplasmosis is the most common cause of posterior uveitis in many countries, occurring subsequently after systemic infection with an obligate, intracellular, protozoan parasite, Toxoplasma gondii (T. gondii) [1]. Members of the feline family are the organism’s definitive hosts while humans and hundreds of other species may serve as intermediate hosts. Transmission to humans occurs by three principal routes: ingestion of raw or inadequately cooked infected meat, ingestion of oocysts (the environmentally resistant form of the organism defecated by cats that is found in cat litter and soil), and via vertical transmission [2]. 

T. gondii has a high affinity for the retinal microvascular endothelium with the retina being the primary site of infection in the eye [1,3]. Primary toxoplasmic retinochoroiditis can be defined as creamy-white exudative focal retinochoroiditis not associated with pre-existing retinochoroidal scars in either eye [4]. Fundus lesions of ocular toxoplasmosis at presentation can be characterized as primary or recurrent and active or inactive. 

Chorioretinal lesions in Toxoplasma infection of the eye can occur either due to congenital or acquired infection [5]. Worldwide, the major victimized demographic in ocular toxoplasmosis continues to be congenitally infected fetuses and newborns, up to 95% of which may show retinochoroiditis that is mostly bilateral and recurrent [6,7]. Unilateral lesions are more common with acquired toxoplasmosis [8]. Lesions in immunocompetent individuals are less severe as compared to those in immunocompromised individuals [1]. 

Choroidal neovascular membrane (CNVM) is a late complication of ocular toxoplasmosis, mostly occurring in healed, inactive lesions and may be a cause of sudden loss of vision, especially in young patients [1,6,9]. Inflammatory CNVM in active toxoplasmic retinochoroiditis is a rare finding, having recently been reported by Hedge et al. However, factors in favour of an acquired or congenital aetiology of the ocular toxoplasmic lesions were not highlighted in the study [10]. 

We report the clinical presentation, diagnosis, and treatment of an immunocompetent adult with unilateral, primary, active toxoplasmic retinochoroiditis of an acquired aetiology that was complicated by early choroidal neovascularization. This case is from Pakistan, where no literature exists on ocular toxoplasmosis. 

## Case presentation

A 22-year-old, unmarried South Asian female who is a computer operator by profession and who owns 10 unimmunized domestic cats at home since two years presented to our clinic with the complaint of metamorphopsia for four months in the right eye. According to the patient, four months ago, she had developed a headache that lasted one day. The headache had been dull in nature and had subsided on its own. Subsequently, straight grid lines on Microsoft Excel spreadsheets appeared wavy to her when viewed through the right eye. She also had refractive errors in both the right (-1.00 dioptre sphere, DS) and left (-0.50 DS) eyes since three years. On ocular examination, best-corrected visual acuity (BCVA) in the right eye was 6/24. Visual acuity in the left eye was 6/18 with BCVA of 6/6. The anterior segment examination was unremarkable. 

**Figure 1:**
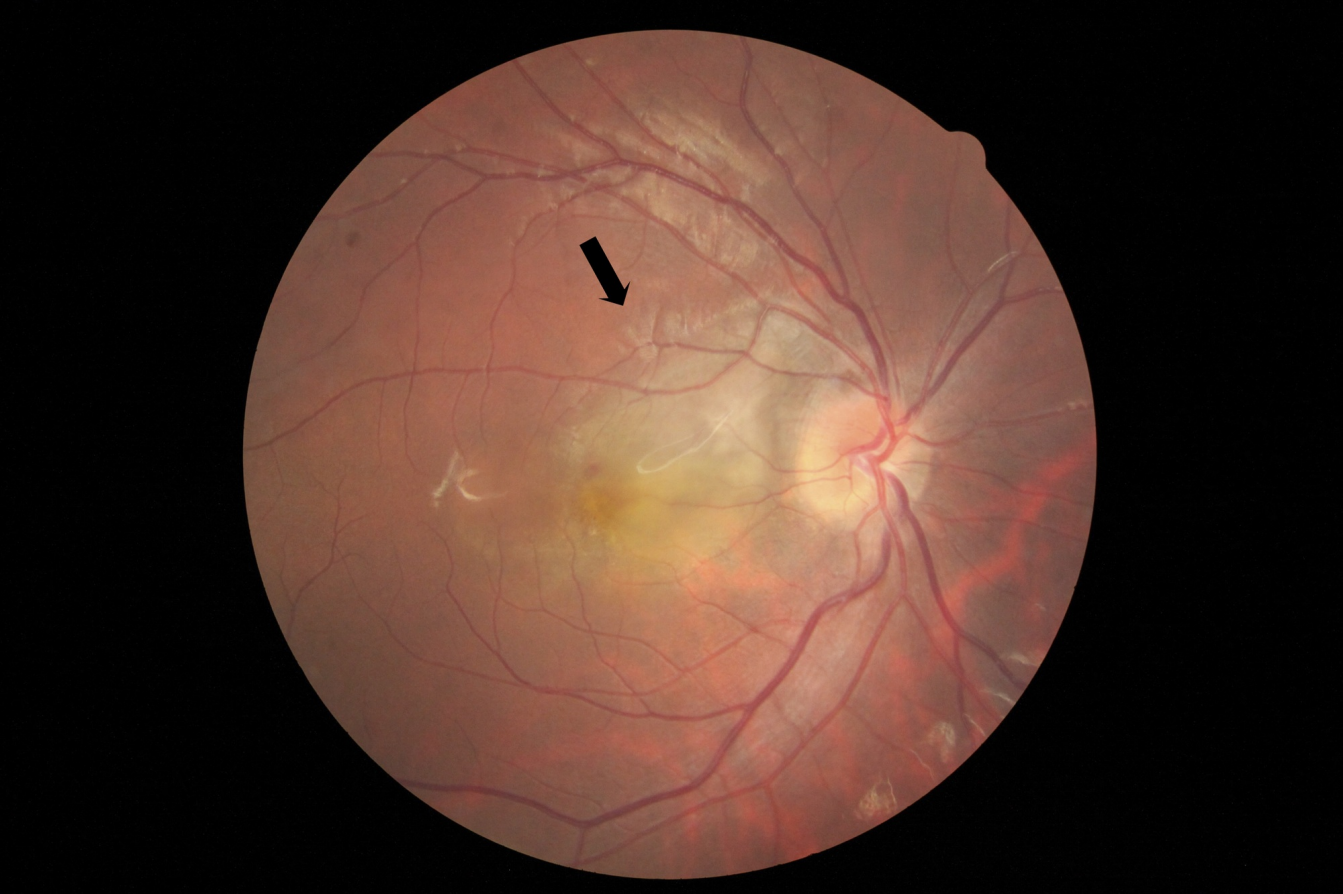
Fundus photograph of the right eye Shows yellow-white active retinitis of two to three disc diameters over the macula.

**Figure 2:**
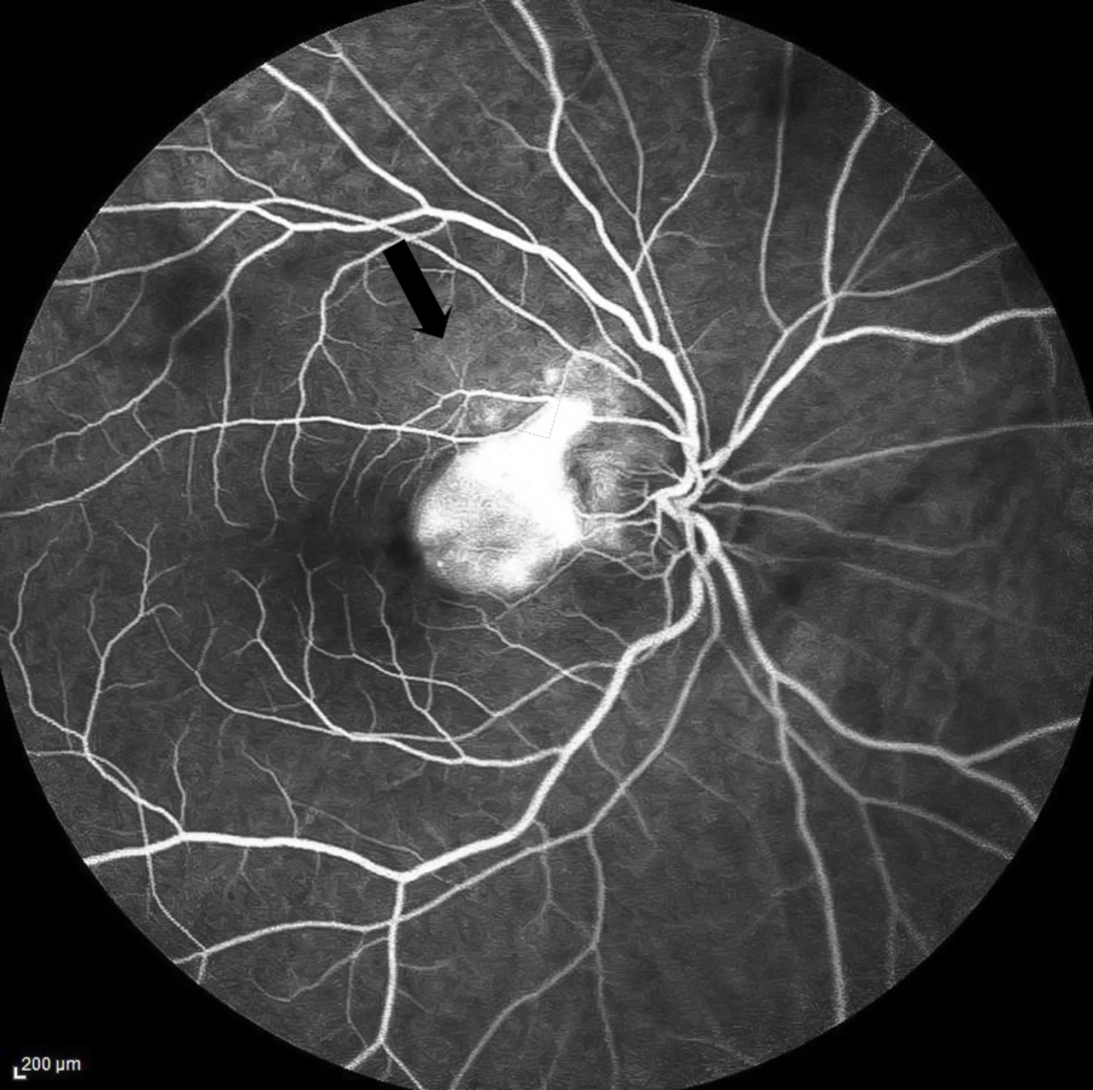
Fundus fluorescein angiography of the right eye in the late phase Shows a hyperfluorescent patch of increasing intensity of two disc diameter temporal to the optic disc which is a choroidal neovascular membrane.

Fundus examination of the right eye revealed 2+ vitreous cells and a slightly elevated area of yellow-white active retinitis of two to three disc diameters (DD) over the macula (Figure 1). Fundus fluorescein angiography (FFA) in the late phase showed CNVM as a hyperfluorescent patch of increasing intensity of 2DD temporal to the optic disc (Figure 2). An axial optical coherence tomography (OCT) scan of the right eye revealed an irregular, thickened surface of the retina with loss of foveal contour and pigment epithelial detachment, findings highly suggestive of choroidal neovascular membrane (Video 1). Optical coherence tomography angiography (OCTA) revealed a patch of abnormally dilated, hyperfluorescent vessels at the level of the outer retina. 

**Video 1:** Axial optical coherence tomography scan of the right eye before treatment Shows retinal pigment epithelial detachment, disruption of Bruch's membrane, subretinal fluid and hyper-reflective material, characteristic of CNVM.

Clinical manifestations of systemic Toxoplasma infection were absent. There was no history of ocular trauma or any comorbid condition. Her past medical and surgical history was insignificant. There was no history of drug use, allergies, addiction, or blood transfusion. She had normal sleep, appetite, and bowel habits. Findings on complete blood count (CBC), erythrocyte sedimentation rate (ESR), and chest X-ray were within the normal range. The serum angiotensin-converting enzyme (ACE) level was normal. Mantoux test was negative and Hepatitis B virus surface antigen (HBsAg), Hepatitis C virus antibodies (HCV-Ab) and Venereal Disease Research Laboratory-Rapid Plasma Reagin (VDRL-RPR) were non-reactive. An enzyme-linked immunosorbent assay (ELISA) of serum for anti-Toxoplasma antibodies showed high titers of immunoglobulin G, while serum immunoglobulin M (IgM) was absent. The late presentation of the patient may explain the absence of serum IgM. 

Therefore, in light of the above findings, a diagnosis of primary acquired Toxoplasma retinochoroiditis with active CNVM was made. Treatment was commenced with a combination of sulfamethoxazole and trimethoprim DS (800 mg/160 mg) twice daily that was continued for 21 days. Two days after antibiotic therapy was begun, oral prednisolone, 25 mg twice daily, was given and gradually tapered over the next three weeks. Intravitreal injection of anti-vascular endothelial growth factor (anti-VEGF) and bevacizumab (1.25mg/0.05ml), was given monthly for three months. 

After three months of treatment, the vision in her right eye improved to 6/18, but on the Amsler grid she still appreciated curved lines. Vitreous cells had disappeared. Axial OCT was repeated that showed that the thickness of the retina had decreased temporal to the fovea, but there was still a dome between the disc and fovea (Video 2). Therefore, currently bevacizumab is being continued and further response monitored.

**Video 2:** Axial optical coherence tomography scan of right eye after three months of treatment Shows decreased thickness of the retina temporal to the fovea.

## Discussion

While Toxoplasma gondii infects up to one-third of the world’s population, South East Asia counts as a region of low seroprevalence for the organism [2,11]. Although Tasawar et al. reported a seroprevalence of T. gondii infection of 29.45% in Southern Punjab, Pakistan, no literature on its nationwide prevalence exists, and ocular toxoplasmosis has not been reported in the region [12]. 

Typical ocular complaints such as those of blurred vision and floaters secondary to vitritis were absent in our patient, who complained of metamorphopsia and sudden loss of vision attributable to macular involvement and CNVM. Ophthalmologists, especially those practising in areas of low seroprevalence, must therefore keep Toxoplasma infection as a differential diagnosis when presented with any of such complaints due to the worldwide distribution of T. gondii. 

The diagnosis of ocular toxoplasmosis is mainly clinical and serology for anti-Toxoplasma antibodies only supports systemic exposure to the organism [9,13]. While the most common presentation of toxoplasmic retinochoroiditis is the presence of a focus of active necrotizing retinochoroiditis at the border of an old retinochoroidal scar, ophthalmologists must also be wary of primary lesions where pre-existing fundal scarring adjacent to the active lesion is absent as in the case of our patient. 

Although most ocular toxoplasmic lesions in adulthood have previously been presumed to be reactivations of congenital disease, recent literature suggests that postnatally acquired infection may be a more important cause of symptomatic ocular toxoplasmosis than congenital infection [1,5,14]. Although the lesions of toxoplasmic chorioretinitis due to congenital and postnatally acquired infection are morphologically indistinguishable, proximity to cats as well as the absence of a previously existing retinochoroidal scar is suggestive of an acquired aetiology in this patient [15]. 

Cotliar and Friedman contend that severe macular toxoplasmosis in the young is associated with choroidal neovascularization and, therefore, any patient with macular toxoplasmosis must be followed up for the development of CNVM [9]. The case of our young patient supports this view, as CNVM was observed at the first presentation of this patient, even before follow-up. 

The combination of pyrimethamine and sulfadiazine has been classically used as anti-Toxoplasma therapy; however, we used a combination of sulfamethoxazole and trimethoprim to treat this patient for its ability delay the onset of recurrences and provide faster resolution of retinochoroiditis and improved visual acuity [13]. With vision-threatening macular involvement, the value of using oral prednisolone, as in this case, cannot be undermined. As intravitreal anti-VEGF therapy minimizes the destruction of the retina and choroid and provides a favourable visual outcome, its use to eliminate CNVM appeared to be a reasonable treatment option in a developing country where photodynamic therapy is expensive and currently unavailable at many centres [16].

## Conclusions

Despite being an unusual complication of a primary active lesion in an immunocompetent adult, CNVM should be considered and investigated by ophthalmologists as a potential occurrence in patients diagnosed with primary Toxoplasma retinochoroiditis. Moreover, ocular toxoplasmosis can have an acquired aetiology instead of a congenital one as in this case. Prompt diagnosis can prevent visual loss in patients. Lastly, the anti-VEGF, bevacizumab, can be used as a therapeutic and cost-effective treatment for CNVM complicating retinochoroiditis caused by Toxoplasma gondii.
